# Computational Methods for the Integrative Analysis of Genomics and Pharmacological Data

**DOI:** 10.3389/fonc.2020.00185

**Published:** 2020-02-27

**Authors:** Jimmy Caroli, Martina Dori, Silvio Bicciato

**Affiliations:** Department of Life Sciences, University of Modena and Reggio Emilia, Modena, Italy

**Keywords:** genomics, pharmacogenomics, integration, bioinformatics, online databases

## Abstract

Since the pioneering NCI-60 panel of the late'80's, several major screenings of genetic profiling and drug testing in cancer cell lines have been conducted to investigate how genetic backgrounds and transcriptional patterns shape cancer's response to therapy and to identify disease-specific genes associated with drug response. Historically, pharmacogenomics screenings have been largely heterogeneous in terms of investigated cell lines, assay technologies, number of compounds, type and quality of genomic data, and methods for their computational analysis. The analysis of this enormous and heterogeneous amount of data required the development of computational methods for the integration of genomic profiles with drug responses across multiple screenings. Here, we will review the computational tools that have been developed to integrate cancer cell lines' genomic profiles and sensitivity to small molecule perturbations obtained from different screenings.

## Introduction

**Figure 1:**
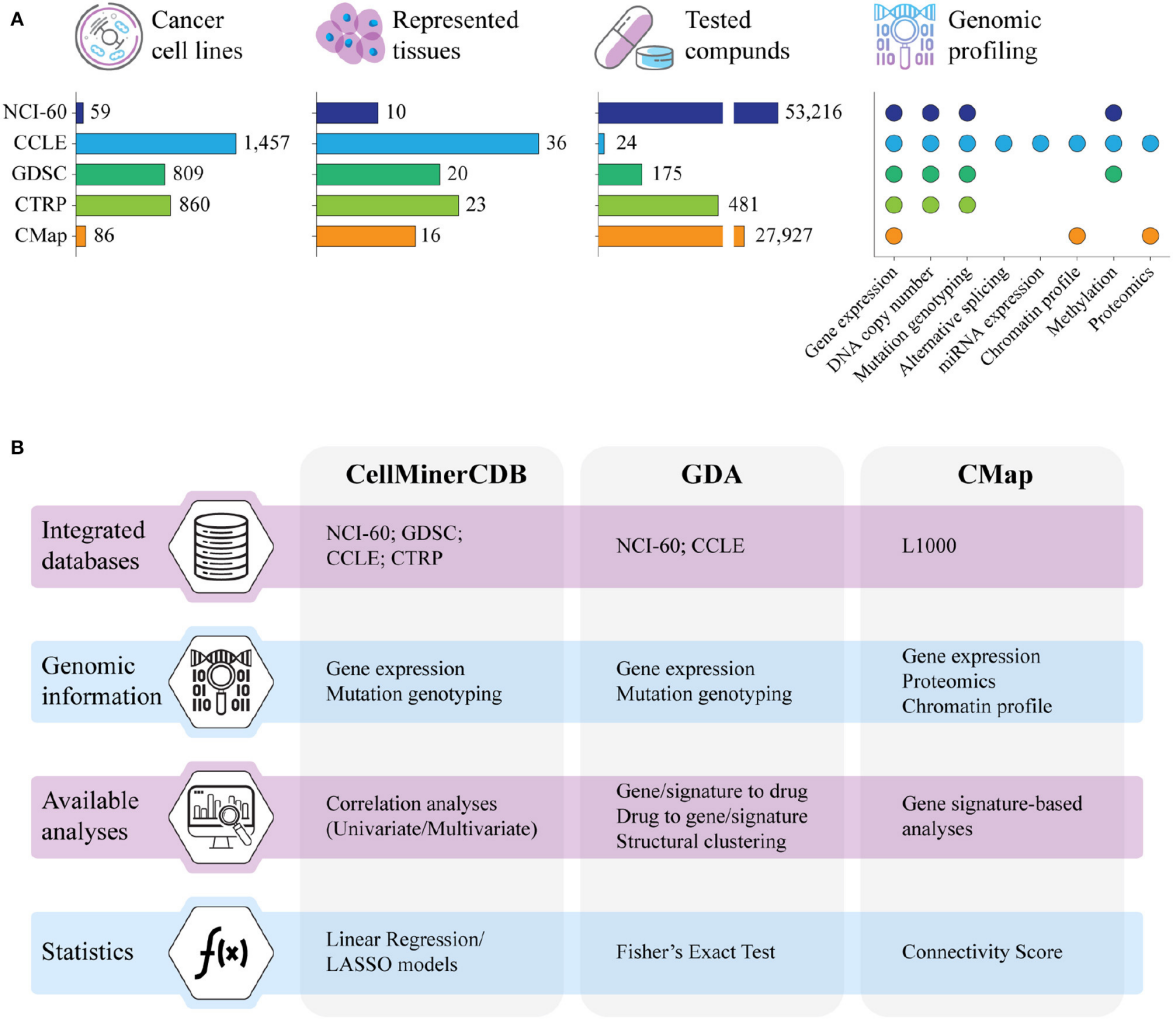
**(A)** Summary of the major resources of pharmacogenomics data in terms of number of cell lines with genomic data, represented tissues, tested compounds, and type of genomic information. NCI-60; CCLE, Cancer Cell Line Encyclopedia; GDSC, Genomics of Drug Sensitivity in Cancer; CTRP, Cancer Therapeutic Response Portal; CMap, Connectivity Map. **(B)** Main characteristics of CellMinerCDB, Genomics and Drugs integrated Analysis portal (GDA), and CMap, the computational resources for the integrative analysis of pharmacogenomics data that are described in this review. LASSO, least absolute shrinkage and selection operator.

Clinical responses to cancer treatment are strongly influenced by the patient's genomic landscape, pushing modern therapeutics toward a more personalized approach (1). To this end, despite their inability to reflect many aspects of a drug's behavior in the human body, cancer cell lines have been the most widely used models to explore the molecular basis of drug activity. Indeed, since the NCI-60 project, several major screenings of unite genetic profiling and drug testing have been created to investigate how genomic portraits can shape cancer response to therapy. These efforts required the definition of integrated frameworks that, leveraging on high-throughput technologies and computational methods, addressed the identification of genomic factors of cancer vulnerability associated with drug sensitivity. The NCI-60 project (https://dtp.cancer.gov/discovery_development/nci-60/) has been the first extensive screening of a massive number of chemical compounds (>50,000) on a well-defined set of cancer cell lines (60 across nine different tumoral tissues) (2, 3). Building on the NCI-60 approach, several other projects investigated the interplay between genomic backgrounds and responses to drug treatment in cancer cell lines (Figure 1A). All cancer cell line screenings basically adopt two approaches. In the first strategy, the molecular profiles of untreated cells and their response to various compounds are investigated in parallel to assess or predict how the molecular portraits determine intrinsic cell sensitivity and resistance to drugs or potential drugs. In the second, cell lines are profiled both before and after treatment to assess how their expression profiles respond to perturbation by the various agents tested. In particular, the Cancer Cell Line Encyclopedia (CCLE, https://portals.broadinstitute.org/ccle) project fully characterized the molecular profiles of more than 1,000 untreated cancer cell lines along with their response to a panel of 24 Food and Drug Administration (FDA)-approved drugs (4–6). Similarly, the Genomics of Drug Sensitivity in Cancer (GDSC, https://www.cancerrxgene.org) and the Cancer Therapeutics Response Portal (CTRP, http://portals.broadinstitute.org/ctrp/) linked genomic features of more than 800 cancer cell lines to their sensitivity to hundreds of chemical compounds comprising FDA-approved drugs, clinical candidates, and small molecules (7–11). Conversely, the Connectivity Map (CMap) and its recent development, L1000 (CLUE, https://clue.io), profiled cancer cell lines before and after the treatment with several chemical compounds and genomic perturbagens, retrieving gene signatures directly associated to their administration (12–14). Although these screenings share a similar experimental pipeline, most of the produced data are heterogeneous and lack concordance in terms of investigated cell lines, tested compounds, and genomic information. In this review, we will describe some computational tools for the integrative analysis of data from different pharmacogenomics resources.

## Integrative Analysis of Genomics and Pharmacological Data

Inspired by the NCI-60 project, several collaborative efforts scaled up the number of cancer cell lines investigated in pharmacogenomics studies from the original 60 to more than 1,400, planning to reach over 10,000 publicly available cancer models in the near future (15). The massive amount of genomic and drug response data generated by these screenings are commonly collected in databases that, through dedicated web portals, provide direct insights into potential interactions between the analyzed cancer cell lines and the tested drugs. These databases are commonly equipped with computational resources specifically designed for the navigation and the analysis of the pharmacogenomics data, as for instance GDSCTools (16), CellMiner (17), Enrichr (18), L1000 Viewer (19), PharmacoGx, and PharmacoDB (20, 21), and the recently deployed RING (22). However, most of these tools are database specific and have limited capabilities in integrating data obtained from different screenings. This limitation is mostly due to the heterogeneity of data provided by the various studies, with drug tests not standardized across projects and genomic profiling not always available for the entire panel of cell lines. In addition, data are often unbalanced, with experiments comprising a high number of cell lines screened on few drugs (e.g., CCLE and GDSC) and, vice versa, screenings of large pools of chemical compounds performed on small cohorts of cancer cell lines (as in the NCI-60). Finally, while genomic data are rather homogeneous and can be easily integrated across studies after removing batch effects, pharmacological data derived from distinct experimental designs must be kept separate as they are profoundly different in terms of analytical assays, tested drug concentration, and retrieved inhibitory potential (23, 24). Despite these intrinsic limitations, several approaches have been proposed for the integrative analysis of genomics and pharmacological data collected from different screenings (Figure 1B). In particular, CellMinerCDB combines genomic profiles from NCI-60, CCLE, GDSC, and CTRP with the pharmacological data provided by the NCI-60 screening (25); the Genomics and Drugs integrated Analysis portal (GDA) integrates pharmacological data derived from the NCI-60 with the genomic information of NCI-60 and CCLE (26); and the CMap enables the investigation of the L1000 data through the correlation of gene lists and transcriptional signatures modulated by the drug treatment (12, 14, 27).

### CellMinerCDB: Integrative Cross-Database Genomics and Pharmacogenomics Analyses

CellMinerCDB (https://discover.nci.nih.gov/cellminercdb/) expands the analysis power of CellMiner, the original NCI-60 analysis tool, with the integration of the cancer cell line data from the Sanger/Massachusetts General Hospital GDSC, the Broad/Novartis CCLE, and the Broad CTRP (25, 28). The integrated database comprises all molecular profiles of almost 1,400 different cancer cell lines, together with drug activity for more than 20,000 compounds. The guiding element, used to link pharmacological information to genomic data from different sources, is the set of common cancer cell lines between the NCI-60 and the other resources, with 55 NCI-60 lines shared with GDSC, 44 with CCLE, and 671 in common between CCLE and GDSC. CellMinerCDB performs correlation analyses to investigate and visualize relationships between the drug activity of a compound and the specific profile of a selected molecular feature across all the available cell lines (univariate analysis). In addition, linear regression methods are implemented for the integrative analysis of multiple identifiers (multivariate analysis). The confidence of the associations is assessed by statistical analyses conducted through a basic linear regression model or using least absolute shrinkage and selection operator (LASSO). An interesting feature of CellMinerCDB is the possibility to compare patterns associated to either drug activity or molecular data via the *Compare Pattern* function of the univariate analysis search. This analysis allows the identification of genomic determinants of drug response, as exemplified by the connection found between the expression of Schlafen 11 (SLFN11) and the response to several DNA-targeted anticancer drugs as platinum derivatives, topoisomerase inhibitors, and poly (ADP-ribose) polymerase (PARP) inhibitors (25).

### Genomics and Drugs Integrated Analysis

GDA (gda.unimore.it/) is a web-based tool designed for the integrative analysis of drug response, mutations, and gene expression profiles derived from the NCI-60 consortium and the CCLE (26, 29). GDA comprises 73 cancer cell lines shared by NCI-60 and CCLE and treated with 50,816 compounds and integrates the drug response data from the NCI-60 screening with the mutations and genomic information derived from both CCLE and NCI-60. GDA allows four different types of analyses, namely, *from drug to gene, from gene to drug, from signature to drug*, and *from drug to signature*. Pharmacological and genomic data can be queried to identify drugs correlated to gene mutations (from gene to drug), gene mutations associated to drug responses (from drug to gene), and drugs associated to active gene signatures (from signature to drug). Starting from a drug correlated to gene mutations, gene expression profiles can be used to identify genes differentially expressed in cell lines sensitive to the selected compound. The statistics behind GDA is based on drug response data. Basically, all pairs of cell lines and drugs are defined as responsive if the relative sensitivity is smaller than two standard deviations of the left tail of the distribution of all relative sensitivities, and non-responsive otherwise. Based on genomic data, cell lines are classified as mutant if treated with the compound and carrying the selected set of mutations and as wild type if treated with the compound but without the specific set of mutations. Given these classifications, compounds are ranked using a score defined by the fraction of responsive in mutant multiplied by the fraction of non-responders in wild type. This score ranks each drug based on the enrichment of responsive in the mutant group. The statistical significance of this ranking is computed using a one-tailed Fisher's exact test for the enrichment of responsive in mutant as compared to non-responsive in wild type, given the number of non-responsive in mutant and responsive in wild type. Results are accessible through interactive graphical representations and tables and can be directly fed to external tools as Enrichr for functional annotation (18). When used to identify compounds able to inhibit the proliferation potential of cancer cell lines with aberrant nuclear YAP/TAZ activation, GDA retrieved imatinib analogs and statins as potentially active drugs. Following GDA indications, *in vitro* studies demonstrated that the combination of statins with dasatinib, an imatinib analog enhances YAP/TAZ nuclear exclusion, is able to block YAP/TAZ transcriptional activity, and is much more active in inducing apoptosis in different tissues (29).

### Connectivity Map and the CMap Linked User Environment

CMap (https://www.broadinstitute.org/connectivity-map-cmap) was one of the first computational resources developed for the investigation of connections between transcriptomics and drug-induced perturbations (12). As extensively reviewed in Musa et al. (30), the goal of CMap is to identify drug or disease-associated gene signatures correlating with transcriptomics changes induced by the administration of drugs or chemical compounds (31, 32). The original project comprised the gene expression profiling of three cancer cell lines before and after the treatment with 164 different small molecules, obtaining drug-associated gene signatures for each cell line. This initial version has been recently scaled up through the L1000 Assay Platform, a method to analyze the expression levels of 978 selected landmark transcripts (assayed with 1,058 probes, including 80 controls) that have been shown to be sufficient to recover more than 80% of the information relative to the full transcriptome (14). This new approach translated into the screening of 86 different cancer cell lines using 27,927 unique perturbagens, including 19,811 small molecules and 7,494 genetic perturbations (consisting of overexpression or knockdown of different genes associated with human diseases or biological pathways). This large-scale screening finally resulted in a collection of 476,251 gene expression signatures that can be analyzed through the CMap Linked User Environment (CLUE, https://clue.io). In CLUE, the Query tool allows to input a gene signature (i.e., a list of genes upregulated and downregulated) and search for perturbagens (chemical and/or genetic) that induce a similar (or opposite) expression profile in the treated cells. The statistical significance of the association is assessed through a connectivity score that takes into account the strength of the similarity between the query and the induced signature as compared to the enrichment of all other signatures in the database (14). This approach proved its efficacy in the identification of a novel inhibitor for the serine-threonine kinase CSNK1A, an enzyme essential in specific subtypes of myelodysplastic syndrome and acute myeloid leukemia. Starting from the loss of function signature of CSNK1A1, authors searched CMap for compounds mimicking the loss of this kinase and identified one compound (BRD-1868) with a high connectivity score relative to this signature. Further enzymatic assays confirmed both the binding between BRD-1868 and CSNK1A1 and its inhibitory effect on enzymatic activity (14). From its first publication, CLUE has been expanded to include also proteomics analysis ranging from expression arrays to histone modification signatures.

## Concluding Remarks

Efforts to decipher the molecular mechanisms of cancer stimulated scientists to explore the interconnection between the genomic landscape of cancer models and their response to drug treatments. This resulted in large pharmacogenomics screenings that, with the advent of high-throughput technologies, generated large amounts of genomics and pharmacological data. However, the integration of these precious information is still challenging due to the variable type and number of drugs and cancer cell lines that have been screened by the various projects and the heterogeneous assays used for drug testing in the different studies (23, 24, 33–35). Despite these intrinsic difficulties, several computational approaches have been developed for the integrative analysis of genomics and pharmacological data. Their application allowed to discover several new connections between drug sensitivity and genomic backgrounds, enabling the potential repurposing of commercially available drugs to cancer treatment (36–38). However, these computational resources, although proven effective, still suffer the limitations of the original studies as the sparsity of the drug and cell interaction matrices, the effective impossibility to merge drug response data across different screenings, and the criticalities of cancer cell lines as a reliable cancer model (39–41). To this end, the project for a Patient-Derived Model Database (PDMB) launched in 2012 by the NCI might represent a potential breakthrough as genomic and drug response data directly collected from patients and patient-derived xenografts (PDXs) will reproduce more accurately the cancer disease and its environment than any cell line model (42). Furthermore, while novel experimental models are generating more accurate data, advanced computational methods are under development to enhance the analytical potential of existing algorithms. As recently discussed (43–45), artificial intelligence approaches as network-based models, deep-learning frameworks, and machine-learning techniques are increasingly applied to investigate pharmacogenomics connections and drug repositioning. These methods can be effective not only for data integration but also to predict new interactions and applications of already approved drugs (46–48). In summary, computational approaches for the integration of genomic and pharmacological data have the potential to become crucial for the systematic identification of new biomarkers of drug sensitivity and the discovery of novel anticancer drugs on the basis of specific genetic abnormalities, as long as reliable cellular models and highly curated data become available.

## Author Contributions

JC and SB conceived the project. JC, MD, and SB wrote and revised the manuscript.

### Conflict of Interest

The authors declare that the research was conducted in the absence of any commercial or financial relationships that could be construed as a potential conflict of interest.
